# From Molecular Biology to Novel Immunotherapies and Nanomedicine in Uveal Melanoma

**DOI:** 10.3390/curroncol31020058

**Published:** 2024-02-01

**Authors:** Kamil J. Synoradzki, Natalia Paduszyńska, Malgorzata Solnik, Mario Damiano Toro, Krzysztof Bilmin, Elżbieta Bylina, Piotr Rutkowski, Yacoub A. Yousef, Claudio Bucolo, Sandrine Anne Zweifel, Michele Reibaldi, Michal Fiedorowicz, Anna M. Czarnecka

**Affiliations:** 1 Environmental Laboratory of Pharmacological and Toxicological Research, Mossakowski Medical Research Institute, Polish Academy of Sciences, 5 Pawinskiego Str., 02-106 Warsaw, Poland; ksynoradzki@imdik.pan.pl; 2 Faculty of Medicine, Medical University of Warsaw, 02-091 Warsaw, Poland; natalia.paduszynska@onet.eu (N.P.); m.solnik98@gmail.com (M.S.); 3 Department of Soft Tissue/Bone Sarcoma and Melanoma, Maria Sklodowska-Curie National Research Institute of Oncology, 5 Roentgen Str., 02-781 Warsaw, Poland; elzbieta.bylina@pib-nio.pl (E.B.); piotr.rutkowski@pib-nio.pl (P.R.); 4 Chair and Department of General and Pediatric Ophthalmology, Medical University of Lublin, 1 Chmielna Str., 20-079 Lublin, Poland; toro.mario@email.it; 5 Eye Clinic, Public Health Department, Federico II University, Via Pansini 5, 80131 Naples, Italy; 6 Research and Development Centre Novasome Sp. z o.o., 51-423 Wrocław, Poland; kbilmin@wp.pl; 7 Department of Clinical Trials, Maria Sklodowska-Curie National Research Institute of Oncology, 02-781 Warsaw, Poland; 8 Department of Surgery (Ophthalmology), King Hussein Cancer Centre, Amman 11941, Jordan; yyousef@khcc.jo; 9 Department of Biomedical and Biotechnological Sciences, University of Catania, Via S. Sofia 97, 95123 Catania, Italy; claudio.bucolo@unict.it; 10 Department of Ophthalmology, University Hospital Zurich, 8091 Zurich, Switzerland; sandrine.zweifel@usz.ch; 11 Faculty of Human Medicine, University of Zurich, 8032 Zurich, Switzerland; 12 Department of Surgical Sciences, Eye Clinic Section, Citta della Salute e della Scienza, Turin University, 10122 Turin, Italy; michele.reibaldi@unito.it; 13 Small Animal Magnetic Resonance Imaging Laboratory, Mossakowski Medical Research Institute, Polish Academy of Sciences, 5 Pawinskiego Str., 02-106 Warsaw, Poland; 14 Department of Experimental Pharmacology, Mossakowski Medical Research Institute, Polish Academy of Sciences, 5 Pawinskiego Str., 02-106 Warsaw, Poland

**Keywords:** uveal melanoma, biomarkers, immunotherapy, theranostics, nanomedicine

## Abstract

Molecular biology studies of uveal melanoma have resulted in the development of novel immunotherapy approaches including tebentafusp—a T cell–redirecting bispecific fusion protein. More biomarkers are currently being studied. As a result, combined immunotherapy is being developed as well as immunotherapy with bifunctional checkpoint inhibitory T cell engagers and natural killer cells. Current trials cover tumor-infiltrating lymphocytes (TIL), vaccination with IKKb-matured dendritic cells, or autologous dendritic cells loaded with autologous tumor RNA. Another potential approach to treat UM could be based on T cell receptor engineering rather than antibody modification. Immune-mobilizing monoclonal T cell receptors (TCR) against cancer, called ImmTAC TM molecules, represent such an approach. Moreover, nanomedicine, especially miRNA approaches, are promising for future trials. Finally, theranostic radiopharmaceuticals enabling diagnosis and therapy with the same molecule bring hope to this research.

## 1. Introduction

Uveal melanoma arises from the pigment-producing cells in the iris, ciliary body, and choroid. Its annual incidence is estimated to be around 5–6 cases per million people, but is the most common primary intraocular malignancy in adults. The average age at diagnosis is around 55 years old. The frequency of its occurrence varies depending on race and geographical latitude. The incidence is highest among the Caucasian race (98% of all cases) and at higher latitudes. In Mediterranean countries, there are 2 new cases per 1 million inhabitants per year, while in Scandinavian countries, it is 8–11 per 1 million inhabitants. In the United States, an average of 4.3 new cases per year per 1 million people occur [1,2,3,4]. Over the past few decades, mortality rates have remained stagnant, exceeding 40% for patients who develop systemic diseases and succumb within a decade of diagnosis. Upon metastasis of the disease, life expectancy diminishes to less than one year [4,5,6].

## 2. Methodology

Databases Pubmed, Scopus, and Google Scholar were used. We defined the following keywords as a string of search terms: “uveal melanoma”, “uveal melanoma” AND “molecular biology”, “uveal melanoma” AND “therapy”, “immunotherapy” OR “ICI” OR “Immune checkpoint inhibitor”, “bispecific antibodies”, “cell-based therapies”, “CAR-T” AND “uveal melanoma”, “tebentafusp”, “theranostics”, “nanomedicine” OR “nanoparticles” OR “photosensitizers”, “photodynamic therapy” AND “uveal melanoma”, “targeted cancer treatments”. The search was modified according to the mentioned databases. Language restrictions included literature described in English and Polish.

For clinical trials, we searched two databases: “www.clinicaltrials.gov” and “https://euclinicaltrials.eu/”, with no restrictions on race, place, age, or language.

## 3. Molecular Biology of Uveal Melanoma

The development of uveal melanoma is primarily correlated with specific genetic aberrations including chromosome 3 complete monosomy, 6 disomy, and 8q gain or 8p loss, as well as the expression of the human melanoma black 45 (HMB45) antigen, the S-100 protein, Melan-A (also known as the melanoma antigen recognized by T cells 1/MART-1), melanocyte-inducing transcription factors (MITFs), tyrosinase, vimentin, and sex-determining region Y-Box 10 (SOX10), which were detected in immunohistochemistry [7,8]. The predominant mutation occurs at the Q209 position, with 45% of mutations involving GNAQ and 32% involving GNA11. Less commonly, mutations impact the R183 position, with 3% in GNAQ and 2% in GNA11. These mutations lead to a modification in amino acids and do not occur simultaneously [9]. The GNAQ and GNA11 genes are responsible for encoding heterotrimeric Gq-proteins, which play a role in connecting transmembrane receptors to intracellular pathways. The activation of these genes is viewed as an initial signal in the carcinogenesis of uveal melanoma [10]. Other events in UM development include mutations in BAP-1 and SF3B1 genes. Loss or point mutations in BAP1 occur in more than 80% of metastatic uveal melanoma cases. These events are linked to reduced rates of disease-free survival (DFS) and overall survival (OS). Mutations in the BAP1 gene most often result in the early termination of the BAP1 protein and may affect its ubiquitin carboxyl-terminal hydrolase domain, thereby modifying its deubiquitinase activity. Because BAP1 has interactions with multiple proteins and signaling pathways, including the tumor suppressor BRCA1 gene, it plays a vital role in preserving genome stability and DNA damage response [11,12,13]. Around 10–20% of uveal melanoma cases display mutations in the splicing factor 3B subunit 1 (SF3B1) gene. Situated on chromosome 2q33, the SF3B1 gene is responsible for coding a subunit of the spliceosome, a sizable complex engaged in the processing of precursor mRNA. SF3B1 holds a vital function in ensuring accurate splicing by retaining pre-mRNA and determining the splicing site. The X-linked eukaryotic translation initiation factor 1A protein (EIF1AX) is mutated in about 15% of UM cases. This gene locus is found on chromosome 10. The X-linked eukaryotic translation initiation factor 1A protein is a key player in overseeing the initiation of protein translation. Mutations in EIF1AX can cause the mis-selection of start sites, resulting in the inhibited translation of canonical transcripts or potentially elevating the expression of oncogenes [8,13,14,15,16,17]. Again, *BAP1*, *SF3B1*, and *EIF1AX* mutations are mutually exclusive. Mutations in genes including GNAQ, GNA11, SF3B1, EIF1AX, BAP-1, and PRAME are important in assessing prognoses. Epigenetic mechanisms regulated by non-coding RNAs (ncRNA) including microRNAs and long non-coding RNAs are also deregulated in UM, including miR-513a-5p, miR-182, miR-211, miR-137, miR-20a, and miR-27a [7,8,13,18].

Investigations of tumor-driving cell cycle and epigenetic pathways involving the above-mentioned genes have led to novel targeted therapies. Compounds like monoclonal antibodies or small molecules affect the downstream signaling of activated Gαq/11, targeting the factors crucial for UM pathways: the protein kinase C (PKC)/mitogen-activated protein kinase (MAPK) or phosphatidylinositol-3 kinase (PI3K)/mTOR/AKT and the IGF-1/IGF-1R pathway, which inhibit proteins like YAP (yes-associated protein), focal adhesion kinase (FAK), PARP, bromodomain and extra-terminal (BET), Brahma-associated factor complexes (BAF), or HDAC inhibitors. Preclinical or clinical trials are currently being undertaken to evaluate compounds that target mentioned proteins and pathways [19].

Extending diagnostics with genetic abnormalities in UM cells significantly increases the scope of information, precision, and accuracy of diagnosis and generates further challenges for the effective use of obtained data. Currently, the role of genetic mutations and circulating miRNAs in uveal melanoma is being investigated in the NCT05179174 trial run by the Ophthalmology Clinic of the “Policlinico-Vittorio Emanuele” University Hospital in Catania, Italy; the Ophthalmology Clinic of the University of Turin in Italy; as well as the Department of General and Paediatric Ophthalmology, Medical University of Lublin in Poland. All patients enrolled in the study had blood samples examined for the presence of ctDNA with GNA11 and GNAQ gene mutations and the expression of multiple microRNAs: miR-506-514 cluster, hsa-miR-592, and hsa-miR-199a-5p, using the digital PCR droplet system.

An interesting study employing gene expression profiling (GEP) enabled us to divide UM into two molecular subtypes. US subtypes are referred to as class 1 and class 2 and differ by a subset of 15 gene expressions. The class 1 expression profile is a good prognostic biomarker and is found in approximately 66% of cases. On the contrary, the class 2 profile is a poor prognostic biomarker [20]. Recently, Opa-interacting protein 4 (PRAME)—cancer-testis antigen 130 expressed in UM—has been defined as a new biomarker. This biomarker improves the prognostic specificity of the 15-gene GEP profile prognostic specificity [21]. Moreover, PRAME may also be considered a novel immunotherapy target as it is not expressed in normal cells. Monoclonal antibodies and cytotoxic T lymphocytes reactive against PRAME may become a new therapeutic option and are currently being investigated [22,23].

## 4. Current Therapy of UM

**Figure 1:**
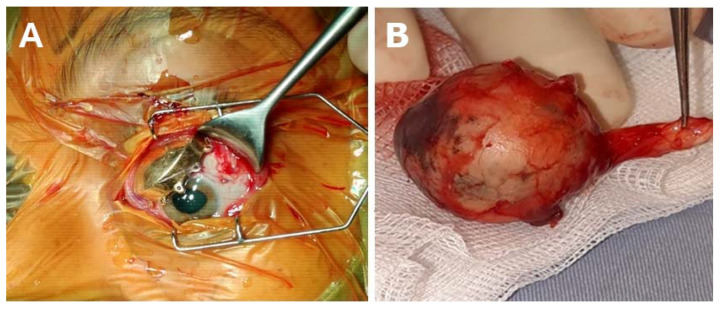
Main Treatment options for choroidal melanoma. Small and medium choroidal melanoma can be treated with radioactive plaque therapy (**A**), while very large tumors generally require enucleation (**B**).

Therapeutic approaches undertaken after the diagnosis of uveal melanoma (UM) and recommended by the National Comprehensive Cancer Network (NCCN) guidelines include radiotherapy (brachytherapy, fractionated stereotactic irradiation, proton beam irradiation) and surgical treatment (local resection, endo-resection, or enucleation) (Figure 1) [24]. Treatment goals encompass eradicating the tumor, maintaining both the eye and visual function, and enhancing overall survival. The spectrum of treatment options ranges from local resection for small and slow-growing tumors to enucleation for large tumors, with no potential for visual recovery. The most comprehensive conservative approach involves episcleral brachytherapy or charged-particle radiotherapy. These methods have demonstrated excellent local control, with a recurrence rate and distant metastasis of about 5% to 20% over five years and survival rates comparable to those of enucleation [5,25,26]. In the recent evolution of intraocular melanoma treatment, there has been a significant shift away from enucleation. Instead, methods such as brachytherapy (iodine-125, ruthenium-106, palladium-103, or cesium-131 plaque brachytherapy), proton beam radiotherapy, stereotactic irradiation or radiation surgery (LINAC, Gamma Knife, CyberKnife), transcleral local resection, transretinal resection, vitreoretinal (VR) surgery, and diode laser phototherapy have taken precedence. Radiotherapy, phototherapy, and local tumor resection are also often used in combination [5,27,28,29,30]. Radiation therapy is currently the most used technique for the treatment of UM, with brachytherapy isotope plaques emitting gamma radiation being used in most ophthalmology centers [31,32]. Among brachytherapy approaches, ruthenium-106 plaque radiotherapy was confirmed to be effective in the treatment of thick and large UM with a high globe preservation rate [33]. Stereotactic radiosurgery with gamma rays is being used to save adjacent structures [34,35] as, during proton beam therapy, tantalum rings are positioned at the tumor margin to delineate the radiation border [36]. In addition to the above, globe-preserving treatment options are transpupillary thermal therapy and photodynamic therapy [37,38]. Other techniques such as laser and photodynamic therapy, transpupillary thermotherapy, photocoagulation, or cryotherapy are also in use based on multidisciplinary team (MDT) decisions [1,24,39,40,41].

Currently, the Institut Curie in Paris is conducting a phase II trial devoted to patients with large UM that evaluates endoresection of the UM tumor scar or transpupillary thermotherapy when endoresection is not feasible after proton beam therapy (NCT02874040). Extensive and advanced tumors are treated with radical methods such as surgical enucleation of the globe, exenteration (removal of an eye and ambient tissue), and uvectomy (selective removal of anterior uveal tract tumors) [1,42,43]. A surgical technique (enucleation) is recommended for tumors too large for brachytherapy, difficult to treat with radiation alone, extensive extraocular extension, or optic nerve involvement [24]. For local treatment, the current trial is evaluating the safety and efficacy profile of hypofractionated stereotactic linear accelerator radiotherapy (NCT00872391). In this study, 10 stereotactic linear accelerator radiotherapy fractions with six Gy per fraction at the 80% isodose for the planning target volume are used and up to 155 patients are to be enrolled.

At the same time, metastatic uveal melanoma is a challenging disease to treat. The most common site of UM metastases is the liver. Hepatic metastases, which occur in approximately 95% of patients with metastatic uveal melanoma, often lead to fatalities in almost all cases. In a large study by Cleveland Clinic, liver metastasis was shown to occur 27 months after uveal melanoma diagnosis (interquartile range 13–46 months). The spread of uveal melanoma to the liver is a significant factor contributing to the severity of the disease and its poor prognosis. Treatment options for metastatic uveal melanoma with liver involvement are limited, and the condition remains a considerable challenge in clinical management [44]. The currently approved therapy for metastatic UM is tebentafusp treatment, as described below in the immunotherapy section. At the same time, liver-directed therapies are in development.

The surgical removal of liver metastases may be an option for some patients if the tumors are limited in number and location. However, surgery is not always feasible, especially if the metastases are widespread or if the patient’s overall health is compromised [45]. Other liver-directed therapies are hepatic artery infusion (HAI) [46] and transarterial chemoembolization (TACE) [47]. The efficiency of chemotherapy using cytotoxic agents in liver metastatic lesions is insignificant [31,43]. Novel modifications of chemoembolization are currently being studied (Table 1). At Thomas Jefferson University, hepatic metastases are currently being carried out with 300 mg of carmustine (BCNU) in ethiodized oil in a phase II trial (NCT04728633). Arterial chemoembolization (TACE) with the infusion of BCNU dissolved in ethiodized oil (Lipiodol^®^) is followed by an injection of gelatin sponge (Gelfoam^®^). Lipodol is also known as ethiodized oil, which is poppy seed oil. Lipodol is claimed to deposit in the tumor when injected into the liver artery and to have prolonged retention in liver tumors (https://www.guerbet.com/, accessed on 27 December 2023). In this trial, patients are treated once every 4 weeks (Q4W) for bilobar disease or once every 7 weeks (Q7W) for unilobar disease.

For liver metastases, Y90 radioembolization and isolated and percutaneous hepatic perfusion are also used [36]. Transarterial hepatic immunoembolization as well as selective internal radiation therapy are in development. The first immunoembolization trials were attempted with included granulocyte-macrophage colony-stimulating factors (GM-CSFs; sargramostim) [48]. The localized trial of Thomas Jefferson University (NCT01473004) confirmed the efficacy of Sir-Spheres^®^ and yttrium-90 microspheres administered intrahepatically. In this trial, spheres were administered once for each involved lobe in 4-week intervals. Trials of liver transplantation for UM (NCT01311466) were not successful, with patients having short disease-free survival and short overall survival from the time of relapse as well as from the time of transplantation [49]. Moreover, multiple trials are ongoing. The Charité University Medicine Berlin phase II trial is ongoing to evaluate the effect of transarterial radioembolization with yttrium-90 microspheres (SIRT) and transarterial chemoembolization with cisplatin (DSM-TACE) (NCT02936388). For liver metastases, pressure-enabled hepatic artery infusion of SD-101—a TLR9 agonist—is also being investigated in the USA (NCT04935229). SD-101 doses should be delivered via the hepatic artery with the TriNav device. The intra-tumoral pressure (ITP) targeted system is claimed to significantly increase the therapeutic delivery of the drug (https://trinavinfusion.com/ accessed on 27 December 2023). At the same time, multiple systemic treatment trials are ongoing (Table 1). Two compounds, binimetinib (MEK1 and MEK2 kinase inhibitor) and crizotinib (ALK kinase inhibitor), are currently being investigated in clinical trials (NCT02223819, NCT03947385) in patients with metastatic UM melanoma. A phase II nonrandomized, single-arm, multicenter study of sitravatinib in combination with tislelizumab is recruiting subjects with metastatic uveal melanomas and liver metastases (NCT05542342).

## 5. Novel Immunotherapy Approaches

**Figure 2:**
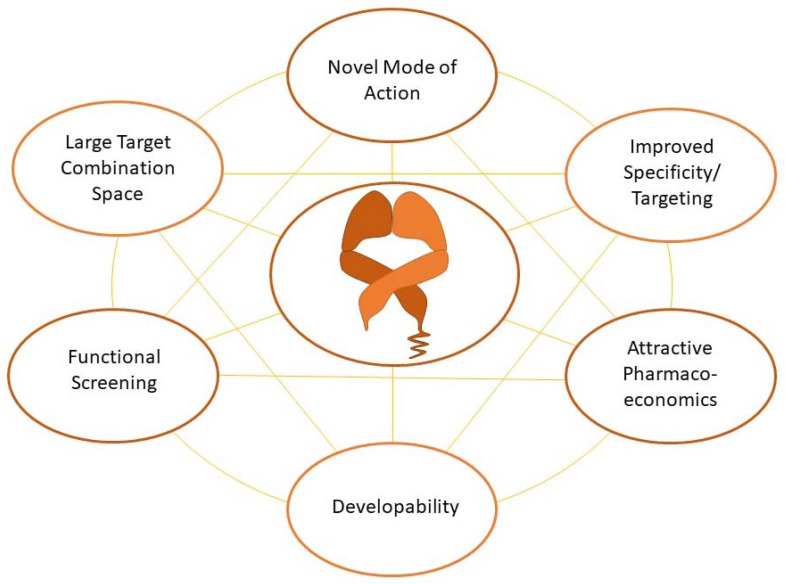
Advantages of Novel Immunotherapy Approaches.

Biological differences between cutaneous melanoma and uveal melanoma significantly impact the efficacy of immunotherapy. In addition to the immune privilege of the eye, uveal melanoma is characterized by a lower number of mutations: low TMB (tumor mutation burden) and low neoantigen load, as well as a highly immunosuppressive tumor microenvironment [50,51]. The suppression of the immune response by UM cells results from the secretion of interleukins IL-2 and IL-10 and macrophage polarization to the M2 phenotype (with immunosuppression activity); UM cells could also deplete tryptophan (essential for lymphocyte activation) through the upregulation of IFN-gamma-inducible indoleamine 2,3-dioxygenase (IDO) [52,53,54,55]. UM has the propensity to spread to the liver, and the liver microenvironment is rich in growth factors, including IGF-1 and HGF and chemokines, i.e., CXCL12, with a slow blood flow. In addition, in liver metastases, a high number of CD4^+^ cells are present, as well as myeloid-derived suppressor cells (MDSCs), while CD8^+^ cells are scarce [56]. All these features favor metastasis formation, UM progression, and immunotherapy resistance. Improving the efficiency of UM immunotherapy (Figure 2) should involve novel targets and immune response activation mechanisms [51].

Potential drug targets enabling the inhibition of immunosuppression in UM are the tyrosine-based inhibitory motif domain (TIGIT) or lymphocyte activation gene 3 (LAG3). These new antigens should be considered as potential candidates for UM patients. TIGIT is a receptor expressed on lymphocytes. It interacts with CD155 expressed in tumor cells or antigen-presenting cells (APCs) and, as a consequence, inhibits T and NK cell function [57]. Immunohistochemical studies reveal a large number of TIGIT-positive cells in primary and metastatic UM [58,59]. LAG3 is a receptor expressed in T cells (including CD4+, CD8+, and Tregs), dendritic cells, and NK cells [60]. LAG3 regulates T cell activation, proliferation production of cytokines, and activity [61]. Dominated T cells (CD8+) at primary tumor sites express LAG3 rather than PD1 or CTLA4 [53].

Bispecific antibodies (bsAb) that target two independent epitopes or antigens represent the next step in UM treatment [62]. Bispecific T cell-redirecting antibodies (TRBAs) bind the antigen on the cancer cell and the second antigen, a component of the T cell receptor complex. Such co-binding bypasses antigen presentation through the major histocompatibility complex pathways and results in cancer cell apoptosis. Two types of TRBA are produced. The first class of TRBAs are large IgG-like bispecific antibodies with extended pharmacokinetics that may be dosed infrequently. Second-class TRBAs are small, short-half-life, bispecific antibodies that include bispecific T cell engagers (BiTEs) that require continuous dosing.

Bispecific T cell engagers (BiTEs) are molecules that are small-sized, flexible, and highly antigen-specific. BiTE molecules are built by two antibody variable fragments joined by linker peptides. One antibody-derived fragment has specificity antitumor-associated antigens (TAAs); the second has, for example, anti-CD3 specificity (T cell receptor complex—a group of molecules that are necessary for T cell activation). Bispecific T cell engagers, in consequence facilitate immune synapses, activate T cells and lead to tumor cells dead [63]. Recently, bispecific antibodies targeting human c-MET have also been developed [64].

Bifunctional checkpoint inhibitory T cell engagers (CiTEs) are larger molecules than BiTEs and combine T cell redirection to tumor cells with immune checkpoint inhibitory function (a part of CiTEs molecule inhibits the PD–1/PD–L1 axis) [63]. The therapeutic activity of this type of molecule (T cell redirection with a restricted blockade of PD-1/PD-L1) was assessed against acute myeloid leukemia (AML). The synergistic effect of checkpoint blockade and the activation of T cells result in high cytotoxicity in vitro (also in patients’ cells) and without adverse events in in vivo models [65].

**Figure 3:**
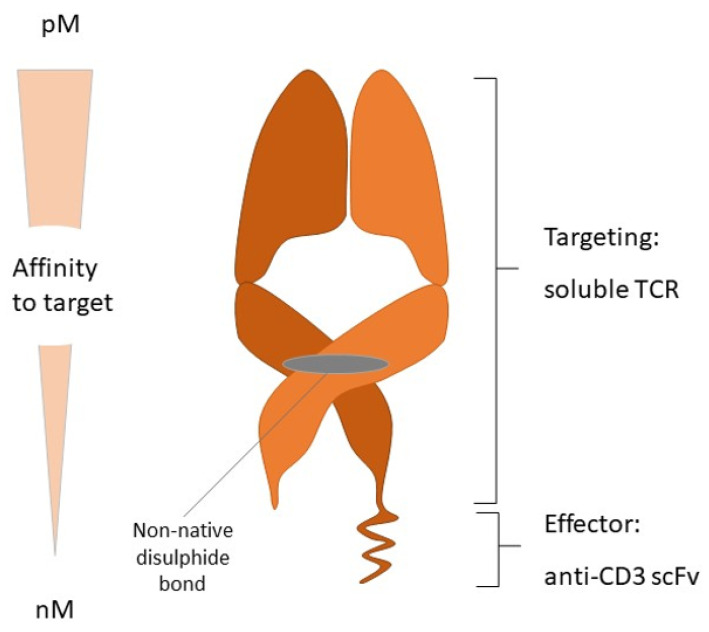
Immune-mobilizing monoclonal T cell receptors (TCRs) against cancer structure.

Another potential approach to treat UM could be based on T cell receptor engineering rather than antibody modification. Immune-mobilizing monoclonal T cell receptors (TCRs) against cancer, called ImmTAC molecules, represent one approach. These particles (Figure 3) are built by a soluble T cell receptor joined with an anti-CD3 single-chain variable fragment. The T cell receptor part targets cells presenting tumor neoantigens or intracellular antigens presented by HLA. The anti-CD3 domain recruits immune cells, mainly (CD3 (+)) T cells [66,67].

**Tebentafusp** is a drug in the class of ImmTAC described above. It is a bispecific, soluble TCR therapeutic. Tebentafusp is an affinity-enhanced TCR fused to anti-CD3 designed to redirect T cells to gp100+ in UM cells. It is a bispecific fusion protein of an engineered high-affinity T cell receptor that presents a peptide-HLA complex on the target cell surface. In detail, tebentafusp consists of a restricted T cell receptor and is specific for the glycoprotein 100 peptide YLEPGPVTA. This construct is further artificially fused to an anti-CD3 single-chain variable fragment. Tebentafusp induces the release of cytokines from T cells and their activation and promotes the trafficking of T cells from the blood into the tumor. As a result, it converts cold tumors into hot tumors. When tebentafusp binds through CD3-specific peptide-HLA complexes on the target UM cell surface, it recruits and activates polyclonal T cells. As a consequence, a release of cytokines and cytolytic mediators against target melanoma cells from the recruited T cells occurs. Tebentafusp was shown to be effective in UM, with overall survival (OS) at 1 year in 73% of the tebentafusp group and 59% in the control group and risk ratio (HR) for death = 0.51 (95% confidence interval [CI] = 0.37 to 0.71; *p* < 0.001) [68,69,70]. The Food and Drug Administration (FDA) has approved tebentafusp for the treatment of HLA-A*02:01 positive adult patients with non-resectable or metastatic uveal melanomas.

**Figure 4:**
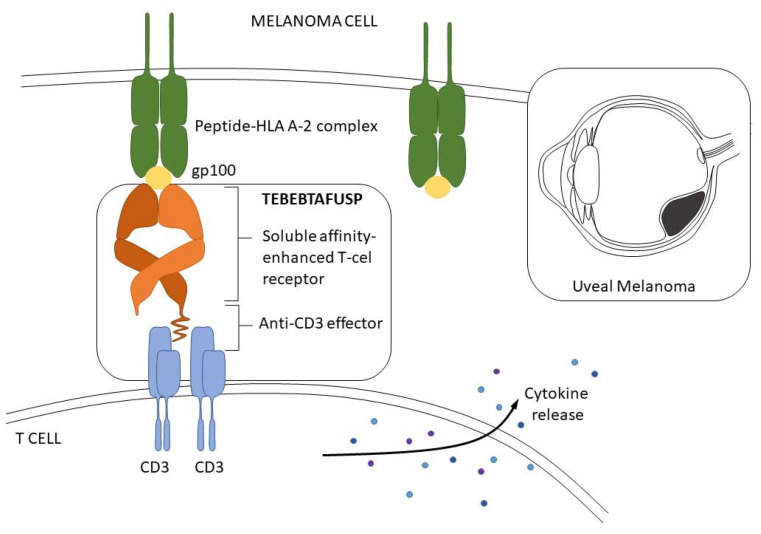
Mechanism of action.

The most famous example of ImmTAC molecules is tebentafusp (Figure 4), but other molecules are under investigation in clinical trials. IMCnyesoa is a fusion protein against tumors expressed (NY-ESO-1) and/or LAGE-1A antigens connected with a single-chain variable fragment specific to the T cell surface antigen CD3. Clinical trials that have used this molecule are included in Table 1. Some trials like NCT03515551 are not recruiting and are awaiting results, while another trial (NCT04262466) is ongoing, and the estimated study completion date is February 2026. This investigation refers to the IMC-F106C molecule, which is also of the ImmTAC type and targets the tumor-associated antigen PRAME [67].

## 6. Combination Immunotherapy in Development

Increasing attention is focused on the use of immunotherapy for uveal melanomas, although the eye is considered an immunologically privileged structure. It should be noted that cancer cells use many of these mechanisms, and some even mimic them, to avoid elimination by the immune system at the sites of metastases. These include the expression of many particles with immunosuppressive activity against T lymphocytes or NK cells, the ability to inactivate complements, inhibit proliferation, and the induction of T cell apoptosis via PD-L1 (programmed death ligand-1) [55]. Immune checkpoint inhibitors (ICIs) are monoclonal antibodies that interact with molecules like programmed cell death 1 (PD-1), programmed cell death 1 ligand 1 (c), or cytotoxic T lymphocyte antigen 4 (CTLA-4); so, these are molecules that allow cancer cells to remain unnoticed by infiltrating lymphocytes. Clinical trials and studies have investigated the use of anti-PD-1 antibodies in uveal melanomas, but results have been mixed. Treatment with anti-CTLA-4 antibodies shows a short median PFS and OS (up to 3.5 and 12.8 months, respectively), with high toxicity. Treatment of anti-PD-1 antibodies shows a longer median overall survival (OS), with lower severe adverse events (AE) [52,71,72]. Pembrolizumab has been studied in Phase II clinical trials in patients with uveal melanomas (stages IIIA, IIIB, IIIC, IV) (NCT02359851), but the benefit was reported only for patients without bulky liver disease. The poor response of UM to ICI treatment likely results from the low expression of PD-L1 molecules in UM tumor cells. The expression of these molecules was observed in 10% and 5.1% of primary tumor site cells and metastatic tumor cells, respectively [73,74]. On the contrary, high mutational load in selected cases is related to the efficacy of checkpoint inhibitors in UM [72]. Combining different checkpoint inhibitors, such as anti-CTLA-4 and anti-PD-1/PD-L1 antibodies, is one approach to enhance the immune response [63]. Multiple clinical trials with active or recruiting statuses referring to ICI UM treatment are currently ongoing. The results of some of the accomplished research have shown that ICIs are not as effective in handling UM as they are for cutaneous melanomas. Combined immunotherapy of anti-PD-1 and CTLA-4 does not exceed 18% overall response rates (ORRs) [72,75]. Clinical trials using immunotherapies are included in Table 1. Interestingly, pembrolizumab with entinostat (histone deacetylase inhibitor), which aims to increase the effects of immunotherapy, is also being evaluated in metastatic disease (NCT02697630) (Table 1). The combination of nivolumab and relatimab (anti-LAG3 antibody; lymphocyte activation gene 3 is expressed in NK cells, B cells, and activated T cells and limits their proliferation and activation) is currently being evaluated in patients with unresectable or metastatic uveal melanomas (NCT03743766) [76]. In addition to that, the synergistic effect of nivolumab and ipilimumab (anti-CTLA 4 antibody, cytotoxic T lymphocyte-associated protein-4, CTLA-4) was investigated as a first-line treatment in patients with metastases (NCT02626962). A different approach involves a combination of immunotherapy with upregulated gene inhibitors in UM, including kinase inhibitors (Table 1) [77]. Another possible target for UM therapy is hepatocyte growth factor receptors/mesenchymal-epithelial transition factors (HGFRs or METs). Their high expression was observed in metastatic uveal melanomas and was correlated with poor prognoses [78].

## 7. Cell-Based Immunotherapies

A specific approach is the infusion of T cells recognizing UM tumor cells. In vitro-based experiments show that activated T cells possess cytolytic activity in primary (MEL202, MEL270, MEL205) and metastatic (OMM2.3) uveal melanoma cell lines [79]. CD8+ cytotoxic T cells constitute the largest population of cells found in primary tumor samples. Natural killer cells (NK), B cells, macrophages, helper/inducer T cells (CD4(+)), and their subset Tregs are also detected, but at low numbers [53,80,81,82,83]. Until now, the adoptive transfer of autologous tumor-infiltrating lymphocytes (TILs) has not been a successful approach in UM treatment [84]. As a result, chimeric antigen receptor (CAR)-modified T cells (CAR-T) have been proposed to be more effective in UM. Patient autologous T cells are isolated and genetically engineered ex vivo with an added constructed receptor. After these modifications, T cells with chimeric antigen receptors are called CAR-T cells. On the transmembrane and intracellular side, they have signal-transducing domains (such as the TCR CD3ζ chain and fragments of 4-1BB and CD28 molecules), and on the extracellular side, they possess a single-chain variable antibody fragment [63].

CAR-T cell therapy was applied in vitro and in vivo with promising results. UM cell lines (92-1 and MEL202 cell lines) were sensitive to CAR-T cells directed against HER2. In in vivo models with 92-1 cells and patient-derived UM cells, tumor rejection was observed. This therapy was also effective against cutaneous and uveal melanoma cells that were resistant to other types of T cell immunotherapy (immune checkpoint blockade or TIL therapy) [85].

The selection of appropriate antigens (or biomarkers) as tumor-associated antigens (TAAs) goes beyond the scope of this review. It should be emphasized that there were several attempts made to search for appropriate TAAs as targets in melanoma cells. Analyses comparing protein expression between skin and uvea melanomas indicate tumor antigens like tyrosinase, melan-A, and SOX10 [18,85,86,87]. Another useful marker for distinguishing cell phenotypes (spindle from epithelioid) in skin melanomas, like p75NTR, is not appropriate in UM spindle cell identification. Markers like HMB-45, HMB-50, tyrosinase, melan-A, and MITF also show differences in expression between these two types of melanomas [88]. In spite of the important role of S100 in the diagnosis of cutaneous melanomas, in UM, it possesses low immunoreactivity [89,90]. Other potentially interesting antigens are those expressed by cancer stem cells (CSCs) like CD133, Pax6, Musashi, Nestin, Sox2, ABCB5, CD166, and ABCB1 transporters that were observed in paraffin-embedded tissues and cell lines [91,92,93]. However, unequivocal characteristics of cancer stem cells in UM are not confirmed. An additional difficulty in the identification of UM TAA antigens is the internal differentiation of tumor cells, as observed in cutaneous melanomas or hair follicles, where expression of the antigens characteristic of melanosomes changes together with the differentiated and undifferentiated states of these cells [87,94]. Despite the above difficulties, clinical trials using CAR-T therapy (NCT03635632) against inter alia uveal melanoma are ongoing. Modified T cells target the disialoganglioside GD2 molecule expressed on tumor cells. The published results of this trial refer to the treatment of high-risk neuroblastoma patients [95].

Another type of cell-based therapy is T cell receptor (TCR)-engineered T cells (TCRSs). This approach uses receptors present on native T cells and adds additional modified T cell receptors. These kinds of modified cells can be used to treat only one type of patient due to their specific genetic background (MHC peptide recognition). The benefit of this therapy is that TCRSs are genetically optimized to recognize epitopes derived from both the cell surface and intracellular targets, including tumor-associated antigens, oncoproteins, tumor-specific neoantigens, and cancer germline antigens expressed in the cytoplasm and nucleus. Such antigens are not detectable by CAR-T due to a different operation module. CAR-T uses antibody-derived elements and is not restricted by MHC molecules [96]. TCRSs have been investigated in clinical trials of metastatic UM. One clinical trial (NCT02743611) used TCR-T therapy for autologous T cells modified to express TCR-targeting PRAME against inter alia metastatic uveal melanomas. The overall status of the study is terminated. Adverse effects of the applied therapy were observed [97]. In the NCT02654821 clinical trial, the transfer of autologous TCR-modified T cells (vector encoding MART-1) was started and a dose-dependent response was observed but, due to toxicity (like dermatitis, uveitis, and ototoxicity), this therapy will likely have a limited use in the future [98].

Natural killer cells (NKs) can also be activated by bispecific antibodies (like T cells). These types of cells are appropriate for therapy because they do not require prior sensitization. Molecules that activate NK cells are composed of a single variable region of an antibody linked to one (bispecific killer engagers, BiKE) or two (tri-specific killer engagers, TriKE) single variable regions of antibodies of different specificity. These elements are joined by a polypeptide linker [63,99]. An example of TriKE is a molecule consisting of single-variable chains against CD16 and CD33 antigens, joined by IL15, to target myeloid malignancies [100]. In the context of NK-based therapies for treating UM, it should be noted that the aqueous humor in the eye contains MIF and TGF-B. Both molecules possess inhibitory effects on cells [101,102]. UM cells produce MIF [103]. Therefore, the effectiveness of potential therapies could also be low. However, it was proven by in vitro experiments that NK cells kill the OCM-3 uveal melanoma cell line [101]. Using NK cells in therapy against UM may be useful at liver metastatic sites. The liver is an organ with the highest concentration of NK cells (up to 50% of all liver lymphocytes) [104]. In animals with a reduction of NK cells, an increase in the volume and number of hepatic micrometastases occurs [105]. The application of intravenous adenovirus-mediated IFN-gamma gene transfer (INF-gamma is a potent activator of NK cells) in mice carrying intraocular melanomas resulted in a reduction in the number of metastatic loci in the liver [106].

## 8. Theranostics: A Step towards Personalized Medicine

The theranostic concept is a hope for development in the field of oncology in the context of precise diagnostics that will enable timely treatment. The appropriate structure of the modified compounds using long-known anticancer substances allows for a highly selective action of these substances at tumor sites while limiting harmful effects on other tissues and persistent side effects for patients [107]. However, once introduced into clinical practice, the high cost of these methods remains an important issue due to their costly, time-consuming, and advanced technology.

A promising strategy that should be mentioned here is to combine noninvasive diagnostic methods with therapy to apply personalized treatment called theranostics. The new imaging methods presented may be utilized in that field because they allow the use of selectively targeted agents (which may be drug-coated) to control their transport to destination and therapy response, and, more importantly, offer the possibility of real-time imaging. However, these methods also have limitations, e.g., reaching the target tissue with the probe. However, various solutions are being developed to ensure probe penetration into tumor tissue, including the use of small particles (nanoparticles, liposomes, micelles), coverage with appropriate particles binding to special receptors, and drug release technology induced by hypoxia, temperature, or specific pH ranges [108].

Due to their beneficial properties, proteins, antibodies, and aptamers could be used as theranostic tools that bind to molecules that are present in excess in tumor cells or their environment, for example, insulin-like growth factor 1 receptor (IGF1R), folate receptor (FR), epidermal growth factor receptor (EGFR), human epidermal growth factor receptor 2 (HER2), vascular endothelial growth factor (VEGF), and integrins [109]. Combining these particles with markers allows for visualization, and the conjugation with anticancer drugs allows for extremely precise and localized therapy. Dal Corso et al. [110] proposed small molecule drug conjugates (SMDC) that contain the Arg-Gly-Asp (RGD) sequence and are ligands for αvβ3 integrins (present in tumor vessels). These conjugates can transport various drugs, e.g., doxorubicin, paclitaxel, cisplatin, or camptothecin, used in systemic chemotherapy, but their toxic effect on the body is significantly reduced. It is also possible to use fluorescent probes activated by extracellular enzymes or intracellular lysosomal transformations [111]. This method improves image quality as the marker is only in the target tissue, and background signals are eliminated. Sun et al. [112] introduced a theranostic prodrug activated with aminopeptidase N (APN), which is a PET probe for diagnosis and therapy. In this case, the toxic substance to cancer cells is melphalan, which acts selectively and saves normal tissues.

An attractive solution in novel theranostic particles is the practical use of a particular characteristic of neoplasms, which maintains a slightly acidic pH (approx. 6.8) [113]. Iron oxide nanoparticles with pH-sensitive magnetic nanogrenades (PMNs) can self-organize under the influence of acidic reactions. Minimal-sized tumors (<3 mm in diameter) can be detected by combined MRI and fluorescence imaging. Ling et al. [113] suggested that pH-targeted photodynamic therapy (where light radiation affects photosensitizers to produce reactive oxygen species) could be used in the treatment of resistant tumors. Kim et al. [114] proposed the use of multifunctional magnetite nanoparticles (AHP @ MNP). In addition to Fe_3_O_4_, they consist of photosensitizers and hyaluronic acid (AHP), and are synthesized from amines. As a result of the surface charge, they can be covered with different coatings. These particles enter tumor cells through CD44 and have a dual nature. They may serve as a tool for magnetic hyperthermia therapy (magnetic nanoparticles convert electromagnetic energy to heat) and photodynamic therapy.

There are also nanoparticles for which activation is enzymatically catalyzed [115]. Such an activation process is possible when the concentration in the tumor environment is increased. The molecule must be adequately designed; it should confirm easy bonding with the catalyst. One of the concepts assumes that the nanoparticle releases the drug after an enzymatic decomposition of the coating structure. The particle surface is properly modified, making it susceptible to enzymatic catalysis, or the drug is placed inside the liposome and released after the carrier’s destruction. Several types of enzymes might be involved in these nanoparticle decompositions, e.g., proteases, lipases, and glycosidases (e.g., cathepsin B, peroxidase, urease, glucose oxidase, α-amylase). This technology, as a part of targeted cancer therapy, allows for enzymatic activity and poses a challenge to better understanding the enzymatic arsenal of tumors.

Kim et al. [116] suggested taking advantage of the increased level of H_2_O_2_ in cancer metastases (as an adaptation to increasing amounts of reactive oxygen species) for imaging and the simultaneous stimulation of the antitumor effect of the transported drug (prodrug 7). In targeted cancer treatments, “prodrug 7” demonstrates a novel approach. Its interaction with hydrogen peroxide (H_2_O_2_) in tumor cells leads to a change in its structure, triggering two key responses: the activation of fluorescence for imaging and the release of the anticancer agent SN-38 (the active metabolite of irinotecan). This targeted mechanism ensures selective and efficient treatment, focusing on H_2_O_2_-rich cancer cells, thereby enhancing the precision and effectiveness of the therapy. Similar properties can be attributed to nanoparticles whose functionality is dependent on tissue hypoxia. Zhou et al. [117] studied an azo derivative (AzP1) irinotecan analog. This compound consists of an inhibitor of topoisomerase I and aryl-azo benzyl alcohol (azo derivative substances undergo a reduction under conditions of low oxygen concentration and serve as hypoxia markers). The visualization of fluorescence also allows for the quantification of the released chemotherapeutic agent.

Several studies are currently underway in this area (although in diseases other than uveal melanoma). Somatostatin-expressing neoplasms such as neuroendocrine tumors, medulloblastoma, meningioma, and neuroblastoma are treated with ^90^Y-DOTATOC, and the usefulness of ^68^Ga-DOTATOC PET/CT is being evaluated in diagnosis and treatment (NCT02441088). Additionally, circulating tumor DNA (cDNA) will be evaluated as a theranostic marker in glioblastomas (NCT03115138). Combining radioactive particles such as ^131^iodine and ^90^yttrium, with appropriate proteins like MIBG or DOTATOC ensures the targeting of drugs to tumor cells and minimizes the side effects of therapy. The concurrent application of ^131^I-MIBG and ^90^Y-DOTATOC in midgut neuroendocrine tumors (NETs) and SPECT/CT control will be performed in one study (NCT03044977). In turn, the study (NCT04769817) will analyze the results of patients previously treated with lutetium 177 (^177^Lu)–PSMA with metastatic castration-resistant prostate cancer (mCRPC) and evaluate the analogy between PSMA and PET/CT images (18F-FDG).

If theranostics for uveal melanomasdevelop in the future, it would be worth adopting immunotherapy (Table 2). Although the development of adequately modified particles and their production is demanding and time-consuming, it will be a promising element of effective cancer therapy [118].

## 9. Nanomedicine

Nanomedicine is a field at the interface of medicine and pharmacy encompassing very small technologies, ranging from 1 to 1000 nm, to treat or diagnose diseases. The use of nanoscale materials allows for an appropriate concentration of a drug at the target site of action. It can be understood as a part of personalized medicine in this sense [119]. Nanomaterials used in medicine and pharmacy can be divided into three main types: polymers, inorganic nanoparticles, and lipid technologies, especially liposomes. These technologies have found their practical application and, currently, there are medicinal products on the market using all the forms mentioned above. One of the first FDA-approved drugs used in oncology were liposomal forms of doxorubicin (Doxil, Janssen) and an *Escherichia coli*-derived conjugate of L-asparaginase with monomethoxypolyethylene glycol for the treatment of acute lymphoblastic leukemia (Oncaspar, Servier) (Mitchell MJ, 2021). The first nanosystem approved for use in photodynamic therapy was verteporfin in liposomal form (Visudyne, Novartis; Bausch and Lomb), which is currently used in AMD and CNV photodynamic therapy as well as for uveal melanoma [120,121]. Nanotechnologies play a crucial role in advancing personalized medicine in oncology. They enhance the delivery of drugs directly to tumor sites by efficiently overcoming biological barriers. Additionally, nanotechnologies are valuable for enhancing the physical and chemical characteristics of drugs, contributing to their increased efficacy in treating cancer. A good example that confirms the advantages of nanomedicine to improve personalized medicine is imatinib, the first drug in the targeted therapy of chronic myeloid leukemia with the expression of the BCR-ABL fusion protein. The increase in drug delivery using nanomaterials has been shown to improve survival by 40% in a mouse leukemia model [122]. Nanomedicine may be particularly important in ophthalmology due to the specific structure of the eye and biological barriers that represent an obstacle to drug delivery [123]. Therefore, it seems that nanomedicine can be very helpful in UM therapy, where overcoming biological barriers of the eye and effective drug delivery to the tumor site are of particular importance. Current nanomedicine research on uveal melanoma uses nanoparticles to deliver cytostatic drugs and genes and increases the effectiveness of brachytherapy or photodynamic therapy. In addition to therapeutic nanoparticles, contrast agents have also been developed, which, given in the form of nanoparticles, increase the quality of the radiological images [124]. One of the most dynamically developing areas of nanomedicine is the delivery systems of miRNA—an important regulator of gene expression and one of the important molecular targets in the development of anticancer therapies. Disturbances of miRNA expression in UM are classified as epigenetic mechanisms responsible for tumorigenesis and metastasis [125]. Preclinical studies have shown that therapeutic miRNAs can inhibit the proliferation of UM tumor cells, reduce their growth, prevent metastasis, and increase susceptibility to radiation therapy. Unfortunately, the stability of miRNAs in vivo is low due to the destructive effect of serum nuclease, among other reasons. This makes it difficult to achieve the right concentration in cancer cells. To overcome this problem and protect against the degradation of miRNA molecules, drug delivery systems based on organic (polymers, liposomes, micelles, etc.) and inorganic nanoparticles of gold, silver, iron oxide, silicon, etc. [126] can be used. The synergistic cytotoxic effect of a type I topoisomerase inhibitor and miRNA was shown in the study by Milain Rois et al. [127]. It turned out that both compounds, conjugated with gold nanoparticles as carriers, showed a stronger carrier cytotoxic effect against human uveal melanoma cells. The authors emphasized that the use of gold nanoparticles as a carrier of miRNA allows to increase internalization into cells and, in the case of a cytostatic drug, solves the problem of its poor solubility in water. This technology increased the anticancer efficacy of both substances [127].

In addition to gold nanoparticles, one of the most commonly used compounds as a drug carrier in nanomedicine is albumin. This approach has also been used in the experimental therapy of uveal melanoma. Albumin-based nanostructures containing AZD8055 (ABN-AZD), a potent inhibitor of mTOR kinase, proved to be selective and showed toxicity only to uveal melanoma cells, while not affecting keratinocyte cells. Moreover, these nanostructures showed excellent in vivo activity, reducing tumor size compared to free AZD8055 in mouse models [128].

In addition to developing completely new therapeutic methods, nanomedicine is also an excellent tool for preparing new formulations and new routes of administration of known anticancer drugs. One such approach concerns an encapsulated lipid nanostructure of sorafenib for the treatment of UM. The developed technology could overcome the biological barriers to the eye and is an important step in the development of topical application of anticancer agents used in UM [129]. Another solution that used increased penetration into eye tissues and extended release was a curcumin-loaded nanoparticle/hydrogel formulation. In addition to favorable pharmacokinetic parameters, the new curcumin formulation showed significant antitumor activity against MP-38 human uveal melanoma cells [130]. Chlorin e6, a photosensitizer used in photodynamic therapy for cancer, is another example of a well-known substance used in the development of a nanoparticle. The authors of one the study proposed an innovative approach to the diagnosis and treatment of UM by using multifunctional chlorin e6 (Ce6) in poly-lactic-co-glycolic acid (PLGA) NPs and wrapping Fe III-tannic acid (Fe III-TA) nanoparticles (FTCPNPs), combining photothermal therapy (PTT) and photodynamic therapy (PDT). FTCPNPs is actually a theranostic, as it not only induces apoptosis of tumor cells through mitochondrial damage, but also works as a MRI contrast agent [131].

It should also be emphasized that apart from their undoubted advantages, nanomaterials can also be dangerous, and the assessment of their safety profile is a challenge for science and regulatory agencies. In the study of Ding et al. [132], it was shown that carbon dot (C-dot) nanoparticles at a certain concentration could increase the growth of uveal melanoma cells in a zebrafish model and nude mouse xenograft. The postulated mechanism of this phenomenon is that C-dot induces a moderate increase in ROS, resulting in the activation of the Akt/mTOR pathway and increasing glutamate metabolism, which can ultimately cause the excessive growth and aggressiveness of UM cells, as well as metastasis [132].

In 2020, at the annual ARVO conference, an interim analysis of a phase 1b/2 clinical trial of AU-a011—novel light-activated nanomedicine to treat UM was published [133]. AU-011, developed by Aura Biosciences, Inc., is a targeted therapy based on a viral carrier and a phototoxic drug. This conjugate irradiated by 689 nm wavelength light causes an increased concentration of singlet oxygen and necrosis of the cancer cell. Furthermore, damaged cells induce an antitumor immune response by releasing damage-associated molecular patterns [134]. The results of this interim analysis are very promising, as tumor growth control was observed in more than 60% of the patients, visual acuity remained unchanged in more than 90% and the safety profile was favorable.

## 10. Conclusions

Personalized uveal melanoma (UM) medicine is a promising and dynamically developing field that covers several aspects such as patient predispositions, diagnostics, clinical management, determination of prognosis, and treatment [6]. Multiple attempts to develop new treatment approaches (Table 2) and clinical trials are on the way. Improved diagnostic methods allow for the detection of specific differences that influence the choice of therapeutic options. The above considerations underline great progress in developing personalized uveal melanoma therapy with the use of nanotechnology. Unfortunately, these findings have not translated into clinical practice. There is an unmet medical need that stimulates research in this area. The use of nanomaterials in personalized uveal melanoma therapy has many benefits and can be a helpful tool to increase the effectiveness of treatment. However, in designing further studies and assessing the effectiveness of a novel therapy, it is also necessary to consider its potential toxic effects and safety issues. Immunological therapies could be applied to uveal melanoma, but more research is needed to assess UM-specific antigens and reduce the immunosuppressive effects of immune cells in the tumor microenvironment.

## Figures and Tables

**Table 1:** Investigational compounds in the treatment of uveal melanoma or metastatic disease.

Evaluated Treatment	Mechanism	Clinical Trial	Status
DYP688	N/A mechanism	NCT05415072	Recruiting
Pembrolizumab 25 MG/1 ML Intravenous Solution	anti-PD-1	NCT05282901	Recruiting
Pembrolizumab Lenvatinib	anti-PD-1 + multiple kinase inhibitor	NCT05308901	Recruiting
Autologous Tumor-Infiltrating Lymphocytes Melphalan Interleukin-2	T cell therapies	NCT04812470	Recruiting
Tebentafusp	ImmTAC molecule	NCT05315258	Recruiting
Vaccination with IKKb-Matured Dendritic Cells	IKKb-matured dendritic cells	NCT04335890	Active, not recruiting
Nivolumab + Relatimab	Anti-PD-1 + anti-LAG-3	NCT03743766	Active, not recruiting
C7R-GD2 ART Cells Cyclophosphamide Fludarabine	T cell therapies	NCT03635632	Active, not recruiting
Tumor-Infiltrating Lymphocytes (TIL)	T cell therapies	NCT03467516	Recruiting
Aldesleukin Autologous CD8+ SLC45A2-specific T Lymphocytes Cyclophosphamide Ipilimumab	T cell therapies + Anti-CTLA-4	NCT03068624	Active, not recruiting
Ipilimumab Nivolumab Melphalanchemosaturation via Percutaneous Hepatic Perfusion	anti-CTLA-4 + anti-PD-1	NCT04283890	Recruiting
Nivolumab	anti-PD-1	NCT03025256	Active, not recruiting
Isolated Hepatic Perfusion Ipilimumab Nivolumab	anti-CTLA-4 + anti-PD-1	NCT04463368	Active, not recruiting
Nivolumab Relatlimab	anti-PD-1 + anti-LAG-3	NCT04552223	Active, not recruiting
Autologous Dendritic Cells Loaded with Autologous Tumor RNA	T cell therapies	NCT01983748	Active, not recruiting
Stereotactic Body Radiotherapy Ipilimumab Nivolumab	radiation anti-CTLA-4 + anti-PD-1	NCT05077280	Recruiting
NovacureOptune Opdivo Yervoy	anti-PD-1 + anti-CTLA-4	NCT05004025	Recruiting
FHD-286	BRG1 and BRM enzymatic inhibitor	NCT04879017	Active, not recruiting
Binimetinib Belinostat	MEK inhibitor + histone deacetylase inhibitors	NCT05170334	Recruiting
SD-101 Nivolumab Ipilimumab	agonist of toll-like receptor 9 (TLR9) + anti-PD-1 + anti-CTLA-4	NCT04935229	Recruiting
Hypofractionated Linearaccelerator Radiotherapy	radiation	NCT00872391	Recruiting
Endoresection Laser Diode	N/A procedure	NCT02874040	Active, not recruiting
Carmustine Ethiodized Oil Transarterial Chemoembolization Medical Device Usage and Evaluation	procedure	NCT04728633	Recruiting
Sunitinib Valproic Acid	tyrosine kinase inhibitor has an effect on the production of GABA	NCT02068586	Active, not recruiting
AU-011 Suprachoroidal Microinjector PDT Laser	photodynamic therapy	NCT04417530	Active, not recruiting
6MHP NeoAg-mBRAF PolyICLC CDX-1140	peptides to stimulate helper T cells/biologic vaccines anti-CD40	NCT04364230	Active, not recruiting
IDE196 Binimetinib Crizotinib	PKC inhibitor + MEK inhibitor + ALK and ROS1 inhibitor	NCT03947385	Recruiting
IOA-244 Avelumab Pemetrexed Cisplatin Ruxolitinib	inhibitor of PI3Kδ + anti-PD-L1 + kinase inhibitors	NCT04328844	Active, not recruiting
APG-115 + Pembrolizumab	binding of the protein inhibitor to MDM2 + anti-PD-1	NCT03611868	Recruiting
Spartalizumab	anti-PD-1, PCD-1	NCT04802876	Recruiting
PV-10 (10% Rosebengaldisodium)	T cell therapies	NCT00986661	Active, not recruiting
LVGN3616 + LVGN6051 + Nab-Paclitaxel LVGN3616 + LVGN6051 + Bevacizumab + Cyclophosphamide LVGN3616 + LVGN6051 + LVGN7409 + Nab-Paclitaxel LVGN3616 + LVGN6051 + LVGN7409 + Bevacizumab + Cyclophosphamide	anti-PD-L1/PD-1 anti-VEGF-A	NCT05075993	Recruiting
KZR-261	protein secretion inhibitor	NCT05047536	Recruiting
SEA-CD40 Pembrolizumab Pemetrexed Carboplatin	anti-CD-40 + anti-PD-1	NCT04993677	Active, not recruiting
Adoptive Therapy with Autologous MC2 TCR T cells	T cell therapies	NCT04729543	Recruiting
IN10018 Cobimetinib	FAK inhibitor + MEK inhibitor	NCT04109456	Recruiting
Defactinib VS-6766	focal adhesion kinase inhibitor + RAF/MEK inhibitor	NCT04720417	Active, not recruiting
Pembrolizumab Stereotactic Radiosurgery	anti-PD-1 + radiation	NCT02858869	Active, not recruiting
Device: Sir-Spheres^®^	radioactive element	NCT01473004	Active, not recruiting
Vorinostat	histone deacetylase inhibitor	NCT01587352	Active, not recruiting
Melphalan/HDS	cytostatic	NCT02678572	Active, not recruiting
Vaccination with IKKb-Matured Dendritic Cells	IKKb-matured dendritic cells	NCT04335890	Active, not recruiting

Data as of 30 November 2023. Clinical trials were searched at https://www.clinicaltrials.gov/ with the terms “uveal melanoma” and “uveal melanoma treatment”.

**Table 2:** Treatment approaches and their mechanism of action.

Treatment Approach	Mechanism of Action
Current therapy	Ionizing radiation, surgical treatment
Bispecific antibodies	Immune response, engagement of immune cells
Monoclonal T cell receptors	T cell receptor cytotoxicity
Bispecific antibodies that include bispecific T cell engagers (BiTEs) Bifunctional checkpoint inhibitory T cell engagers (CiTEs)	T cell receptor cytotoxicity and inhibition of the PD–1/PD–L1 axis
Immune checkpoint inhibitors	Immune checkpoint inhibitory function by inhibiting molecules like programmed cell death 1 (PD-1), programmed cell death 1 ligand 1, and cytotoxic T lymphocyte antigen 4 (CTLA-4)
Cell-based therapies	Chimeric antigen receptor (CAR)-modified T cells (CAR-T) and T cell receptor (TCR)-engineered T cells (TCRS)—T cell receptor cytotoxicity NK cell activation
Theranostic	Visualization of changed area and anticancer activity by substances with known mechanism of action, photosensitization, enzymatic activity, chemotherapeutic agent release, radioactive particle decay
Nanomedicine	Different drug or miRNA delivery systems: organic, inorganic, topical application of anticancer agents, photosensitization

## References

[1] Kaliki S., Shields C.L. (2017). Uveal melanoma: Relatively rare but deadly cancer. Eye.

[2] Singh A.D., Turell M.E., Topham A.K. (2011). Uveal melanoma: Trends in incidence, treatment, and survival. Ophthalmology.

[3] Piperno-Neumann S., Piulats J.M., Goebeler M., Galloway I., Lugowska I., Becker J.C., Vihinen P., Van Calster J., Hadjistilianou T., Proença R. (2019). Uveal Melanoma: A European Network to Face the Many Challenges of a Rare Cancer. Cancers.

[4] Rutkowski P., Romanowska-Dixon B., Markiewicz A., Zieniewicz K., Kozak K., Rogala P., Świtaj T., Dudzisz-Sledź M. (2022). Diagnostic and therapeutic management of patients with ocular melanomas–r—Recommendations of the Polish Society of Oncology. Nowotw. J. Oncol..

[5] Banou L., Tsani Z., Arvanitogiannis K., Pavlaki M., Dastiridou A., Androudi S. (2023). Radiotherapy in Uveal Melanoma: A Review of Ocular Complications. Curr. Oncol..

[6] Jackson S.E., Chester J.D. (2015). Personalised cancer medicine. Int. J. Cancer.

[7] Shields C.L., Say E.A.T., Hasanreisoglu M., Saktanasate J., Lawson B.M., Landy J.E., Badami A.U., Sivalingam M.D., Hauschild A.J., House R.J. (2017). Personalized Prognosis of Uveal Melanoma Based on Cytogenetic Profile in 1059 Patients over an 8-Year Period: The 2017 Harry S. Gradle Lecture. Ophthalmology.

[8] Broggi G., Russo A., Reibaldi M., Russo D., Varricchio S., Bonfiglio V., Spatola C., Barbagallo C., Foti P.V., Avitabile T. (2020). Histopathology and Genetic Biomarkers of Choroidal Melanoma. Appl. Sci..

[9] Griewank K.G., Yu X., Khalili J., Sozen M.M., Stempke-Hale K., Bernatchez C., Wardell S., Bastian B.C., Woodman S.E. (2012). Genetic and molecular characterization of uveal melanoma cell lines. Pigment. Cell Melanoma Res..

[10] Onken M.D., Worley L.A., Long M.D., Duan S., Council M.L., Bowcock A.M., Harbour J.W. (2008). Oncogenic mutations in GNAQ occur early in uveal melanoma. Investig. Ophthalmol. Vis. Sci..

[11] Szalai E., Wells J.R., Ward L., Grossniklaus H.E. (2018). Uveal Melanoma Nuclear BRCA1-Associated Protein-1 Immunoreactivity Is an Indicator of Metastasis. Ophthalmology.

[12] Uner O.E., See T.R.O., Szalai E., Grossniklaus H.E., Stålhammar G. (2021). Estimation of the timing of BAP1 mutation in uveal melanoma progression. Sci. Rep..

[13] Pašalić D., Nikuševa-Martić T., Sekovanić A., Kaštelan S. (2023). Genetic and Epigenetic Features of Uveal Melanoma-An Overview and Clinical Implications. Int. J. Mol. Sci..

[14] Yavuzyigitoglu S., Koopmans A.E., Verdijk R.M., Vaarwater J., Eussen B., van Bodegom A., Paridaens D., Kiliç E., de Klein A. (2016). Uveal Melanomas with SF3B1 Mutations: A Distinct Subclass Associated with Late-Onset Metastases. Ophthalmology.

[15] Furney S.J., Pedersen M., Gentien D., Dumont A.G., Rapinat A., Desjardins L., Turajlic S., Piperno-Neumann S., de la Grange P., Roman-Roman S. (2013). SF3B1 mutations are associated with alternative splicing in uveal melanoma. Cancer Discov..

[16] Decatur C.L., Ong E., Garg N., Anbunathan H., Bowcock A.M., Field M.G., Harbour J.W. (2016). Driver Mutations in Uveal Melanoma: Associations with Gene Expression Profile and Patient Outcomes. JAMA Ophthalmol..

[17] Ewens K.G., Kanetsky P.A., Richards-Yutz J., Purrazzella J., Shields C.L., Ganguly T., Ganguly A. (2014). Chromosome 3 status combined with BAP1 and EIF1AX mutation profiles are associated with metastasis in uveal melanoma. Investig. Ophthalmol. Vis. Sci..

[18] Barbagallo C., Stella M., Broggi G., Russo A., Caltabiano R., Ragusa M. (2023). Genetics and RNA Regulation of Uveal Melanoma. Cancers.

[19] Wei A.Z., Maniar A.B., Carvajal R.D. (2022). New targeted and epigenetic therapeutic strategies for the treatment of uveal melanoma. Cancer Gene Ther..

[20] Field M.G., Durante M.A., Decatur C.L., Tarlan B., Oelschlager K.M., Stone J.F., Kuznetsov J., Bowcock A.M., Kurtenbach S., Harbour J.W. (2016). Epigenetic reprogramming and aberrant expression of PRAME are associated with increased metastatic risk in Class 1 and Class 2 uveal melanomas. Oncotarget.

[21] Field M.G., Decatur C.L., Kurtenbach S., Gezgin G., van der Velden P.A., Jager M.J., Kozak K.N., Harbour J.W. (2016). PRAME as an Independent Biomarker for Metastasis in Uveal Melanoma. Clin. Cancer Res..

[22] Jindal V. (2018). Role of immune checkpoint inhibitors and novel immunotherapies in uveal melanoma. Chin. Clin. Oncol..

[23] Amir A.L., van der Steen D.M., van Loenen M.M., Hagedoorn R.S., de Boer R., Kester M.D., de Ru A.H., Lugthart G.J., van Kooten C., Hiemstra P.S. (2011). PRAME-specific Allo-HLA-restricted T cells with potent antitumor reactivity useful for therapeutic T-cell receptor gene transfer. Clin. Cancer Res..

[24] Rao P.K., Barker C., Coit D.G., Joseph R.W., Materin M., Rengan R., Sosman J., Thompson J.A., Albertini M.R., Boland G. (2020). NCCN Guidelines Insights: Uveal Melanoma, Version 1.2019: Featured Updates to the NCCN Guidelines. J. Natl. Compr. Cancer Netw..

[25] Abrams M.J., Gagne N.L., Melhus C.S., Mignano J.E. (2016). Brachytherapy vs. external beam radiotherapy for choroidal melanoma: Survival and patterns-of-care analyses. Brachytherapy.

[26] Verma V., Mehta M.P. (2016). Clinical Outcomes of Proton Radiotherapy for Uveal Melanoma. Clin. Oncol..

[27] Romano M.R., Catania F., Confalonieri F., Zollet P., Allegrini D., Sergenti J., Lanza F.B., Ferrara M., Angi M. (2021). Vitreoretinal Surgery in the Prevention and Treatment of Toxic Tumour Syndrome in Uveal Melanoma: A Systematic Review. Int. J. Mol. Sci..

[28] Foti P.V., Travali M., Farina R., Palmucci S., Spatola C., Liardo R.L.E., Milazzotto R., Raffaele L., Salamone V., Caltabiano R. (2021). Diagnostic methods and therapeutic options of uveal melanoma with emphasis on MR imaging-Part II: Treatment indications and complications. Insights Imaging.

[29] Jager M.J., Shields C.L., Cebulla C.M., Abdel-Rahman M.H., Grossniklaus H.E., Stern M.-H., Carvajal R.D., Belfort R.N., Jia R., Shields J.A. (2020). Uveal melanoma. Nat. Rev. Dis. Primers.

[30] Binder C., Mruthyunjaya P., Schefler A.C., Seider M.I., Crilly R., Hung A., Meltsner S., Mowery Y., Kirsch D.G., Tehe B.S. (2019). Practice Patterns for the Treatment of Uveal Melanoma with Iodine-125 Plaque Brachytherapy: Ocular Oncology Study Consortium Report 5. Ocul. Oncol. Pathol..

[31] Souto E.B., Zielinska A., Luis M., Carbone C., Martins-Gomes C., Souto S.B., Silva A.M. (2019). Uveal melanoma: Physiopathology and new in situ-specific therapies. Cancer Chemother. Pharmacol..

[32] Jaradat I., Zewar A., AlNawaiseh I., AlRawashdeh K., Khurma S., Mehyar M., Abdeen G., Yousef Y.A. (2018). Characteristics, management, and outcome of patients with uveal melanoma treated by Iodine-125 radioactive plaque therapy in a single tertiary cancer center in Jordan. Saudi J. Ophthalmol..

[33] Mirshahi R., Sedaghat A., Jaberi R., Azma Z., Mazloumi M., Naseripour M. (2022). Ruthenium-106 plaque radiotherapy for uveal melanoma: Analysis of tumor dimension and location on anatomical and functional results. BMC Ophthalmol..

[34] Sikuade M.J., Salvi S., Rundle P.A., Errington D.G., Kacperek A., Rennie I.G. (2015). Outcomes of treatment with stereotactic radiosurgery or proton beam therapy for choroidal melanoma. Eye.

[35] Dunavoelgyi R., Dieckmann K., Gleiss A., Sacu S., Kircher K., Georgopoulos M., Georg D., Zehetmayer M., Poetter R. (2011). Local tumor control, visual acuity, and survival after hypofractionated stereotactic photon radiotherapy of choroidal melanoma in 212 patients treated between 1997 and 2007. Int. J. Radiat. Oncol. Biol. Phys..

[36] Damato B., Kacperek A., Errington D., Heimann H. (2013). Proton beam radiotherapy of uveal melanoma. Saudi J. Ophthalmol..

[37] Rundle P. (2014). Treatment of posterior uveal melanoma with multi-dose photodynamic therapy. Br. J. Ophthalmol..

[38] Shields C.L., Shields J.A., Perez N., Singh A.D., Cater J. (2002). Primary transpupillary thermotherapy for small choroidal melanoma in 256 consecutive cases: Outcomes and limitations. Ophthalmology.

[39] Amaro A., Gangemi R., Piaggio F., Angelini G., Barisione G., Ferrini S., Pfeffer U. (2017). The biology of uveal melanoma. Cancer Metastasis Rev..

[40] Zewar A., Nawaiseh I., Jaradat I., Khzouz J., Alrawashdeh K., Abdeen G., Mehyar M., Khurma S., Yousef Y.A. (2016). Management and Outcome of Uveal Melanoma in a Single Tertiary Cancer Center in Jordan. Turk Patoloji Derg..

[41] Berus T., Halon A., Markiewicz A., Orlowska-Heitzman J., Romanowska-Dixon B., Donizy P. (2017). Clinical, Histopathological and Cytogenetic Prognosticators in Uveal Melanoma—A Comprehensive Review. Anticancer Res..

[42] Barker C.A., Salama A.K. (2018). New NCCN Guidelines for Uveal Melanoma and Treatment of Recurrent or Progressive Distant Metastatic Melanoma. J. Natl. Compr. Cancer Netw..

[43] Chattopadhyay C., Kim D.W., Gombos D.S., Oba J., Qin Y., Williams M.D., Esmaeli B., Grimm E.A., Wargo J.A., Woodman S.E. (2016). Uveal melanoma: From diagnosis to treatment and the science in between. Cancer.

[44] Grossniklaus H.E. (2013). Progression of ocular melanoma metastasis to the liver: The 2012 Zimmerman lecture. JAMA Ophthalmol..

[45] Rowcroft A., Loveday B.P.T., Thomson B.N.J., Banting S., Knowles B. (2020). Systematic review of liver directed therapy for uveal melanoma hepatic metastases. HPB.

[46] Sajan A., Fordyce S., Sideris A., Liou C., Toor Z., Filtes J., Krishnasamy V., Ahmad N., Reis S., Brejt S. (2023). Minimally Invasive Treatment Options for Hepatic Uveal Melanoma Metastases. Diagnostics.

[47] Huppert P.E., Fierlbeck G., Pereira P., Schanz S., Duda S.H., Wietholtz H., Rozeik C., Claussen C.D. (2010). Transarterial chemoembolization of liver metastases in patients with uveal melanoma. Eur. J. Radiol..

[48] Sato T., Eschelman D.J., Gonsalves C.F., Terai M., Chervoneva I., McCue P.A., Shields J.A., Shields C.L., Yamamoto A., Berd D. (2008). Immunoembolization of malignant liver tumors, including uveal melanoma, using granulocyte-macrophage colony-stimulating factor. J. Clin. Oncol..

[49] Dueland S., Solheim J.M., Espen Foss A., Grut H., Hagness M., Line P.-D. (2020). Dismal survival following liver transplantation for liver-only metastases in patients with ocular malignant melanoma. Trends Transplant..

[50] Johansson P.A., Brooks K., Newell F., Palmer J.M., Wilmott J.S., Pritchard A.L., Broit N., Wood S., Carlino M.S., Leonard C. (2020). Whole genome landscapes of uveal melanoma show an ultraviolet radiation signature in iris tumours. Nat. Commun..

[51] Kaštelan S., Antunica A.G., Oresković L.B., Pelčić G., Kasun E., Hat K. (2020). Immunotherapy for Uveal Melanoma —Current Knowledge and Perspectives. Curr. Med. Chem..

[52] Wessely A., Steeb T., Erdmann M., Heinzerling L., Vera J., Schlaak M., Berking C., Heppt M.V. (2020). The Role of Immune Checkpoint Blockade in Uveal Melanoma. Int. J. Mol. Sci..

[53] Durante M.A., Rodriguez D.A., Kurtenbach S., Kuznetsov J.N., Sanchez M.I., Decatur C.L., Snyder H., Feun L.G., Livingstone A.S., Harbour J.W. (2020). Single-cell analysis reveals new evolutionary complexity in uveal melanoma. Nat. Commun..

[54] Oliva M., Rullan A.J., Piulats J.M. (2016). Uveal melanoma as a target for immune-therapy. Ann. Transl. Med..

[55] Niederkorn J.Y. (2012). Ocular immune privilege and ocular melanoma: Parallel universes or immunological plagiarism?. Front. Immunol..

[56] Rossi E., Schinzari G., Zizzari I.G., Maiorano B.A., Pagliara M.M., Sammarco M.G., Fiorentino V., Petrone G., Cassano A., Rindi G. (2019). Immunological Backbone of Uveal Melanoma: Is There a Rationale for Immunotherapy?. Cancers.

[57] Harjunpää H., Guillerey C. (2020). TIGIT as an emerging immune checkpoint. Clin. Exp. Immunol..

[58] Stålhammar G., Seregard S., Grossniklaus H.E. (2019). Expression of immune checkpoint receptors Indoleamine 2,3-dioxygenase and T cell Ig and ITIM domain in metastatic versus nonmetastatic choroidal melanoma. Cancer Med..

[59] Fu Y., Xiao W., Mao Y. (2022). Recent Advances and Challenges in Uveal Melanoma Immunotherapy. Cancers.

[60] Woo S.R., Turnis M.E., Goldberg M.V., Bankoti J., Selby M., Nirschl C.J., Bettini M.L., Gravano D.M., Vogel P., Liu C.L. (2012). Immune inhibitory molecules LAG-3 and PD-1 synergistically regulate T-cell function to promote tumoral immune escape. Cancer Res..

[61] Ruffo E., Wu R.C., Bruno T.C., Workman C.J., Vignali D.A.A. (2019). Lymphocyte-activation gene 3 (LAG3): The next immune checkpoint receptor. Semin. Immunol..

[62] Wang S., Chen K., Lei Q., Ma P., Yuan A.Q., Zhao Y., Jiang Y., Fang H., Xing S., Fang Y. (2021). The state of the art of bispecific antibodies for treating human malignancies. EMBO Mol. Med..

[63] Goebeler M.-E., Bargou R.C. (2020). T cell-engaging therapies—BiTEs and beyond. Nat. Rev. Clin. Oncol..

[64] Schwartz G.S., Oliver S. (2020). Methods of Treating Ocular Cancer Using Anti-Met Antibodies and Bispecific Antigen Binding Molecules that Bind MET.

[65] Herrmann M., Krupka C., Deiser K., Brauchle B., Marcinek A., Ogrinc Wagner A., Rataj F., Mocikat R., Metzeler K.H., Spiekermann K. (2018). Bifunctional PD-1 × αCD3 × αCD33 fusion protein reverses adaptive immune escape in acute myeloid leukemia. Blood.

[66] Damato B.E., Dukes J., Goodall H., Carvajal R.D. (2019). Tebentafusp: T Cell Redirection for the Treatment of Metastatic Uveal Melanoma. Cancers.

[67] Orloff M., Seedor R., Sato T. (2022). Review of bi-specific therapies in uveal melanoma. Cancer Gene Ther..

[68] Zimmer L., Vaubel J., Mohr P., Hauschild A., Utikal J., Simon J., Garbe C., Herbst R., Enk A., Kämpgen E. (2015). Phase II DeCOG-study of ipilimumab in pretreated and treatment-naïve patients with metastatic uveal melanoma. PLoS ONE.

[69] Kummer M., Schuler-Thurner B. (2017). Immunotherapy of Uveal Melanoma: Vaccination Against Cancer. Methods Mol. Biol..

[70] Nathan P., Hassel J.C., Rutkowski P., Baurain J.-F., Butler M.O., Schlaak M., Sullivan R.J., Ochsenreither S., Dummer R., Kirkwood J.M. (2021). Overall Survival Benefit with Tebentafusp in Metastatic Uveal Melanoma. N. Engl. J. Med..

[71] Marseglia M., Amaro A., Solari N., Gangemi R., Croce E., Tanda E.T., Spagnolo F., Filaci G., Pfeffer U., Croce M. (2021). How to Make Immunotherapy an Effective Therapeutic Choice for Uveal Melanoma. Cancers.

[72] Schank T.E., Hassel J.C. (2019). Immunotherapies for the Treatment of Uveal Melanoma—History and Future. Cancers.

[73] Kaunitz G.J., Cottrell T.R., Lilo M., Muthappan V., Esandrio J., Berry S., Xu H., Ogurtsova A., Anders R.A., Fischer A.H. (2017). Melanoma subtypes demonstrate distinct PD-L1 expression profiles. Lab. Investig..

[74] Javed A., Arguello D., Johnston C., Gatalica Z., Terai M., Weight R.M., Orloff M., Mastrangelo M.J., Sato T. (2017). PD-L1 expression in tumor metastasis is different between uveal melanoma and cutaneous melanoma. Immunotherapy.

[75] Pelster M.S., Gruschkus S.K., Bassett R., Gombos D.S., Shephard M., Posada L., Glover M.S., Simien R., Diab A., Hwu P. (2021). Nivolumab and Ipilimumab in Metastatic Uveal Melanoma: Results from a Single-Arm Phase II Study. J. Clin. Oncol..

[76] Yu X., Huang X., Chen X., Liu J., Wu C., Pu Q., Wang Y., Kang X., Zhou L. (2019). Characterization of a novel anti-human lymphocyte activation gene 3 (LAG-3) antibody for cancer immunotherapy. MAbs.

[77] Pereira P.R., Odashiro A.N., Lim L.A., Miyamoto C., Blanco P.L., Odashiro M., Maloney S., De Souza D.F., Burnier M.N. (2013). Current and emerging treatment options for uveal melanoma. Clin. Ophthalmol..

[78] Tanaka R., Terai M., Londin E., Sato T. (2021). The Role of HGF/MET Signaling in Metastatic Uveal Melanoma. Cancers.

[79] Bosch J.J., Iheagwara U.K., Reid S., Srivastava M.K., Wolf J., Lotem M., Ksander B.R., Ostrand-Rosenberg S. (2010). Uveal melanoma cell-based vaccines express MHC II molecules that traffic via the endocytic and secretory pathways and activate CD8 cytotoxic, tumor-specific T cells. Cancer Immunol. Immunother..

[80] Meecham W.J., Char D.H., Kaleta-Michaels S. (1992). Infiltrating lymphocytes and antigen expression in uveal melanoma. Ophthalmic Res..

[81] Biswas S.K., Mantovani A. (2010). Macrophage plasticity and interaction with lymphocyte subsets: Cancer as a paradigm. Nat. Immunol..

[82] Whelchel J.C., Farah S.E., McLean I.W., Burnier M.N. (1993). Immunohistochemistry of infiltrating lymphocytes in uveal malignant melanoma. Investig. Ophthalmol. Vis. Sci..

[83] Lagouros E., Salomao D., Thorland E., Hodge D.O., Vile R., Pulido J.S. (2009). Infiltrative T regulatory cells in enucleated uveal melanomas. Trans. Am. Ophthalmol. Soc..

[84] Chandran S.S., Somerville R.P.T., Yang J.C., Sherry R.M., Klebanoff C.A., Goff S.L., Wunderlich J.R., Danforth D.N., Zlott D., Paria B.C. (2017). Treatment of metastatic uveal melanoma with adoptive transfer of tumour-infiltrating lymphocytes: A single-centre, two-stage, single-arm, phase 2 study. Lancet Oncol..

[85] Fernandes B.F., Odashiro A.N., Saraiva V.S., Logan P., Antecka E., Burnier M.N. (2007). Immunohistochemical expression of melan-A and tyrosinase in uveal melanoma. J. Carcinog..

[86] Das D., Kaur I., Ali M.J., Biswas N.K., Das S., Kumar S., Honavar S.G., Maitra A., Chakrabarti S., Majumder P.P. (2014). Exome sequencing reveals the likely involvement of SOX10 in uveal melanoma. Optom. Vis. Sci..

[87] Casalou C., Mayatra J.M., Tobin D.J. (2023). Beyond the Epidermal-Melanin-Unit: The Human Scalp Anagen Hair Bulb Is Home to Multiple Melanocyte Subpopulations of Variable Melanogenic Capacity. Int. J. Mol. Sci..

[88] Iwamoto S., Burrows R.C., Kalina R.E., George D., Boehm M., Bothwell M.A., Schmidt R. (2002). Immunophenotypic Differences Between Uveal and Cutaneous Melanomas. Arch. Ophthalmol..

[89] Kan-Mitchell J., Rao N., Albert D.M., Van Eldik L.J., Taylor C.R. (1990). S100 immunophenotypes of uveal melanomas. Investig. Ophthalmol. Vis. Sci..

[90] Kan-Mitchell J., Liggett P.E., Taylor C.R., Rao N., Granada E.S., Danenberg K.D., White W.L., Van Eldik L.J., Horikoshi T., Danenberg P.V. (1993). Differential S100 beta expression in choroidal and skin melanomas: Quantitation by the polymerase chain reaction. Investig. Ophthalmol. Vis. Sci..

[91] Thill M., Berna M.J., Grierson R., Reinhart I., Voelkel T., Piechaczek C., Galambos P., Jager M.J., Richard G., Lange C. (2011). Expression of CD133 and other putative stem cell markers in uveal melanoma. Melanoma Res..

[92] Djirackor L., Kalirai H., Coupland S.E., Petrovski G. (2019). *CD*166high Uveal Melanoma Cells Represent a Subpopulation with Enhanced Migratory Capacity. Investig. Ophthalmol. Vis. Sci..

[93] Landreville S., Agapova O.A., Kneass Z.T., Salesse C., Harbour J.W. (2011). ABCB1 identifies a subpopulation of uveal melanoma cells with high metastatic propensity. Pigment. Cell Melanoma Res..

[94] Casalou C., Moreiras H., Mayatra J.M., Fabre A., Tobin D.J. (2022). Loss of ‘Epidermal Melanin Unit’ Integrity in Human Skin During Melanoma-Genesis. Front. Oncol..

[95] Del Bufalo F., De Angelis B., Caruana I., Del Baldo G., De Ioris M.A., Serra A., Mastronuzzi A., Cefalo M.G., Pagliara D., Amicucci M. (2023). GD2-CART01 for Relapsed or Refractory High-Risk Neuroblastoma. N. Engl. J. Med..

[96] Wei F., Cheng X.X., Xue J.Z., Xue S.A. (2022). Emerging Strategies in TCR-Engineered T Cells. Front. Immunol..

[97] https://www.clinicaltrials.gov/.

[98] Rohaan M.W., Gomez-Eerland R., Foppen M.H.G., van Zon M., de Boer R., Bakker N.A.M., Pronk L.M., Sari A., Mallo H.A., van de Wiel B.A. (2019). 1184PD—Results of a phase I trial with MART-1 T cell receptor modified T cells in patients with metastatic melanoma. Ann. Oncol..

[99] Felices M., Lenvik T.R., Davis Z.B., Miller J.S., Vallera D.A. (2016). Generation of BiKEs and TriKEs to Improve NK Cell-Mediated Targeting of Tumor Cells. Methods Mol. Biol..

[100] Vallera D.A., Felices M., McElmurry R., McCullar V., Zhou X., Schmohl J.U., Zhang B., Lenvik A.J., Panoskaltsis-Mortari A., Verneris M.R. (2016). IL15 Trispecific Killer Engagers (TriKE) Make Natural Killer Cells Specific to CD33 Targets While Also Inducing Persistence, In Vivo Expansion, and Enhanced Function. Clin. Cancer Res..

[101] Apte R.S., Sinha D., Mayhew E., Wistow G.J., Niederkorn J.Y. (1998). Cutting Edge: Role of Macrophage Migration Inhibitory Factor in Inhibiting NK Cell Activity and Preserving Immune Privilege. J. Immunol..

[102] Rook A.H., Kehrl J.H., Wakefield L.M., Roberts A.B., Sporn M.B., Burlington D.B., Lane H.C., Fauci A.S. (1986). Effects of transforming growth factor beta on the functions of natural killer cells: Depressed cytolytic activity and blunting of interferon responsiveness. J. Immunol..

[103] Repp A.C., Mayhew E.S., Apte S., Niederkorn J.Y. (2000). Human uveal melanoma cells produce macrophage migration-inhibitory factor to prevent lysis by NK cells. J. Immunol..

[104] Nemeth E., Baird A.W., O’Farrelly C. (2009). Microanatomy of the liver immune system. Semin. Immunopathol..

[105] Dithmar S.A., Rusciano D.A., Armstrong C.A., Lynn M.J., Grossniklaus H.E. (1999). Depletion of NK cell activity results in growth of hepatic micrometastases in a murine ocular melanoma model. Curr. Eye Res..

[106] Alizadeh H., Howard K., Mellon J., Mayhew E., Rusciano D., Niederkorn J.Y. (2003). Reduction of liver metastasis of intraocular melanoma by interferon-beta gene transfer. Investig. Ophthalmol. Vis. Sci..

[107] Langbein T., Weber W.A., Eiber M. (2019). Future of Theranostics: An Outlook on Precision Oncology in Nuclear Medicine. J. Nucl. Med..

[108] Bhujwalla Z.M., Kakkad S., Chen Z., Jin J., Hapuarachchige S., Artemov D., Penet M.F. (2018). Theranostics and metabolotheranostics for precision medicine in oncology. J. Magn. Reson..

[109] Kim H., Kwak G., Kim K., Yoon H.Y., Kwon I.C. (2019). Theranostic designs of biomaterials for precision medicine in cancer therapy. Biomaterials.

[110] Dal Corso A., Pignataro L., Belvisi L., Gennari C. (2016). αvβ3 Integrin-Targeted Peptide/Peptidomimetic-Drug Conjugates: In-Depth Analysis of the Linker Technology. Curr. Top. Med. Chem..

[111] Kobayashi H., Choyke P.L. (2011). Target-cancer-cell-specific activatable fluorescence imaging probes: Rational design and in vivo applications. Acc. Chem. Res..

[112] Sun W., Li M., Fan J., Peng X. (2019). Activity-Based Sensing and Theranostic Probes Based on Photoinduced Electron Transfer. Acc. Chem. Res..

[113] Ling D., Park W., Park S.J., Lu Y., Kim K.S., Hackett M.J., Kim B.H., Yim H., Jeon Y.S., Na K. (2014). Multifunctional tumor pH-sensitive self-assembled nanoparticles for bimodal imaging and treatment of resistant heterogeneous tumors. J. Am. Chem. Soc..

[114] Kim K.S., Kim J., Lee J.Y., Matsuda S., Hideshima S., Mori Y., Osaka T., Na K. (2016). Stimuli-responsive magnetic nanoparticles for tumor-targeted bimodal imaging and photodynamic/hyperthermia combination therapy. Nanoscale.

[115] de la Rica R., Aili D., Stevens M.M. (2012). Enzyme-responsive nanoparticles for drug release and diagnostics. Adv. Drug Deliv. Rev..

[116] Kim E.J., Bhuniya S., Lee H., Kim H.M., Cheong C., Maiti S., Hong K.S., Kim J.S. (2014). An activatable prodrug for the treatment of metastatic tumors. J. Am. Chem. Soc..

[117] Zhou Y., Maiti M., Sharma A., Won M., Yu L., Miao L.X., Shin J., Podder A., Bobba K.N., Han J. (2018). Azo-based small molecular hypoxia responsive theranostic for tumor-specific imaging and therapy. J. Control. Release.

[118] Lecocq Q., De Vlaeminck Y., Hanssens H., D’Huyvetter M., Raes G., Goyvaerts C., Keyaerts M., Devoogdt N., Breckpot K. (2019). Theranostics in immuno-oncology using nanobody derivatives. Theranostics.

[119] Fornaguera C., García-Celma M.J. (2017). Personalized Nanomedicine: A Revolution at the Nanoscale. Journal of Personalized Medicine. Pers. Med..

[120] Vladimir T. (2020). Handbook of Materials for Nanomedicine Lipid-Based and Inorganic Nanomaterials.

[121] Bilmin K., Synoradzki K.J., Czarnecka A.M., Spałek M.J., Kujawska T., Solnik M., Merks P., Toro M.D., Rejdak R., Fiedorowicz M. (2022). New Perspectives for Eye-Sparing Treatment Strategies in Primary Uveal Melanoma. Cancers.

[122] Mitchell M.J., Billingsley M.M., Haley R.M., Wechsler M.E., Peppas N.A., Langer R. (2021). Engineering precision nanoparticles for drug delivery. Nat. Rev. Drug Discov..

[123] Weng Y., Liu J., Jin S., Guo W., Liang X., Hu Z. (2017). Nanotechnology-based strategies for treatment of ocular disease. Acta Pharm. Sin. B.

[124] You S., Luo J., Grossniklaus H.E., Gou M.L., Meng K., Zhang Q. (2016). Nanomedicine in the application of uveal melanoma. Int. J. Ophthalmol..

[125] Barbagallo C., Platania C.B.M., Drago F., Barbagallo D., Di Pietro C., Purrello M., Bucolo C., Ragusa M. (2021). Do Extracellular RNAs Provide Insight into Uveal Melanoma Biology?. Cancers.

[126] Yang C., Wang R., Hardy P. (2021). Potential of miRNA-Based Nanotherapeutics for Uveal Melanoma. Cancers.

[127] Milán Rois P., Latorre A., Rodriguez Diaz C., Del Moral Á., Somoza Á. (2018). Reprogramming Cells for Synergistic Combination Therapy with Nanotherapeutics against Uveal Melanoma. Biomimetics.

[128] Latorre A., Latorre A., Castellanos M., Lafuente-Gómez N., Diaz C.R., Crespo-Barreda A., Lecea M., Cordani M., Martín-Duque P., Somoza Á. (2021). Albumin-based nanostructures for uveal melanoma treatment. Nanomedicine.

[129] Bonaccorso A., Pepe V., Zappulla C., Cimino C., Pricoco A., Puglisi G., Giuliano F., Pignatello R., Carbone C. (2021). Sorafenib Repurposing for Ophthalmic Delivery by Lipid Nanoparticles: A Preliminary Study. Pharmaceutics.

[130] Xie L., Yue W., Ibrahim K., Shen J. (2021). A Long-Acting Curcumin Nanoparticle/In Situ Hydrogel Composite for the Treatment of Uveal Melanoma. Pharmaceutics.

[131] Huang T., Xu X., Cheng C., Wang J., Yang L. (2023). Cooperative phototherapy based on bimodal imaging guidance for the treatment of uveal melanoma. J. Nanobiotechnology.

[132] Ding Y., Yu J., Chen X., Wang S., Tu Z., Shen G., Wang H., Jia R., Ge S., Ruan J. (2021). Dose-Dependent Carbon-Dot-Induced ROS Promote Uveal Melanoma Cell Tumorigenicity via Activation of mTOR Signaling and Glutamine Metabolism. Adv. Sci..

[133] Mruthyunjaya P., Schefler A.C., Kim I.K., Bergstrom C., Demirci H., Tsai T., Bhavsar A.R., Capone A., Marr B., McCannel T.A. (2020). A Phase 1b/2 Open-label Clinical Trial to Evaluate the Safety and Efficacy of AU-011 for the Treatment of Choroidal Melanoma. Investig. Ophthalmol. Vis. Sci..

[134] Kines R.C., Thompson C.D., Spring S., Li Z., de Los Pinos E., Monks S., Schiller J.T. (2021). Virus-Like Particle-Drug Conjugates Induce Protective, Long-lasting Adaptive Antitumor Immunity in the Absence of Specifically Targeted Tumor Antigens. Cancer Immunol. Res..

